# Examining the knowledge level of nurses regarding aseptic non touch technique in nurses: a cross-sectional study

**DOI:** 10.1186/s12912-024-02154-x

**Published:** 2024-07-31

**Authors:** Yilin Chen, Xiuzhu Cao, Chang Liu, Wanting Sheng, Jie Wang, Linfang Zhao

**Affiliations:** 1 Nursing Department, Sir Run Run Shaw Hospital, School of Medicine, Zhejiang University, Hangzhou, Zhejiang Province China; 2 Wenzhou Medical University, Wenzhou, Zhejiang Province China

**Keywords:** Healthcare-associated infections, Aseptic non touch technique, Nurses

## Abstract

**Background:**

Healthcare-associated infections (HAIs) are important indicators of healthcare quality. The aseptic non touch technique (ANTT), a standardised aseptic technique, is a crucial preventative measure against HAIs. However, there is insufficient evidence currently available regarding ANTT awareness and proficiency among nurses. This study aimed to investigate the status of ANTT proficiency in a cohort of nurses and analyse the associated risk factors, with the ultimate goal of providing a reference for promoting the application of ANTT in clinical practice.

**Methods:**

The study population was sampled from nurses working in a tertiary hospital in Zhejiang Province, China, in January 2024. A cross-sectional survey was conducted using a self-designed questionnaire to assess ANTT knowledge. Multivariate linear regression analysis was used to analyse the risk factors influencing ANTT knowledge in nurses.

**Results:**

A total of 458 nurses were included in this study. The cohort had an overall score of 49.7% correct responses on th*e* ANTT knowledge questionnaire, with a mean score of 12.4 ± 2.4 out of 25, and 30.1% of the nurses felt that they did not need training related to ANTT. Multivariate linear regression analysis indicated that professional title, educational level, and the need for ANTT training were independently associated with ANTT knowledge in nurses. Nurses with Master’s degrees and associated chief nurses were observed to have higher levels of ANTT knowledge compared to the rest.

**Conclusions:**

ANTT knowledge is insufficient among nurses in China, and its importance is not widely recognised. Continuous efforts are required to strengthen this knowledge. Constructing sustained, multiform, and stratified training programmes may represent an effective method to strengthen ANTT knowledge among nurses and promote its clinical use.

## Background

Healthcare-associated infections (HAIs) represent a major challenge in the prevention and control of healthcare infections globally. Data from the World Health Organisation in 2023 show that the incidence of HAIs is increasing at a rate of 0.06% per year, with a global incidence of 0.14% [1]. In China, the average prevalence of hospital-acquired infections is currently 1.64%, which is higher than the global average [2]. HAIs, a common complication in hospitals, are one of the leading causes of healthcare-related deaths in hospitalised patients. They pose a significant threat to patient safety and cause major medical and economic burdens. Moreover, 834,000 HAIs in England existed between 2016 and 2017, at a projected cost of £2.7 billion, resulting in 28,500 patient deaths and 7.1 million people occupying hospital-bed-days, which equates to 21% of the annual number of bed-days in all National Health Service hospitals in England [3]. The Seventy-fifth World Health Assembly proposed the Global Strategy on Infection Prevention and Control, which developed a global action plan and monitoring framework for HAIs and proposed the goal of substantially reducing the ongoing risk of HAIs by 2030 [4]. Both China and the United States have identified the prevention of HAIs as one of their top 10 patient safety goals to achieve by 2022 [5, 6].

Aseptic techniques are widely used to prevent infections, and it is crucial that they are theoretically sound and are performed correctly [7]. However, there are certain challenges to their clinical application, including confusion between practical terms (such as “clean” and “sterile”), imprecise definitions, and a lack of unified standards. These issues have resulted in high variability and poor controllability, leading to inconsistent levels of understanding among nurses regarding aseptic techniques and a potential inability to accurately comprehend their most appropriate use cases. The significant workload in many hospital wards has led to aseptic techniques being performed hastily, in quicker and easier manners [7]. A few medical staff mistakenly believe that aseptic techniques are only used in operating rooms [8].

The Aseptic non-touch technique (ANTT) is an aseptic technique with a unique theoretical and practical framework that was developed in the late 1990s [9] and updated in 2010 [10]. ANTT achieves aseptic mainly through a unique method of protecting critical parts and sites [9, 10], and is appropriate for all clinical procedures. ANTT is a vital measure for preventing HAIs [11], thereby ensuring quality and safety in healthcare. ANTT represents the first and only complete clinical practice standard for aseptic technique, and it has been widely recognised by the international community and is used in a variety of areas—including hospitals, communities, and military emergency care [12]. The Association for Vascular Access [13] and the Infusion Nurses Society [14] both believe that ANTT should be regarded as an essential clinical competency to guarantee patient safety, similar to the administration of basic life support.

Despite the widespread recognition of the value of ANTT and its promotion and implementation in clinical practice, it still encounters numerous challenges. The research and application of ANTT are still in their infancy, and the status of its application remains unclear—particularly in China—and few studies have been conducted to explore it. This study aimed to investigate the level of ANTT-related knowledge among clinical nurses working in China and to analyse the risk factors associated with this proficiency. The ultimate goal was to promote the dissemination and implementation of ANTT in China and thereby enhance patient safety outcomes.

## Methods

### Study design

A quantitative cross-sectional design was used for this study.

### Participants

Cluster sampling was used to select the study population. A total of 458 nurses from a large tertiary hospital (a teaching hospital in Zhejiang Province, China that includes medical, surgical, emergency and critical care, and surgical suites, over a total of 26 wards) were enrolled in December 2023. The inclusion criteria were as follows: (1) nurses with a licence, and (2) nurses who gave informed consent and volunteered to participate in the survey. The following groups of nurses were excluded from the study population because of their weaker associations with the hospital: (1) nurses on maternity leave, and (2) nurses currently studying as students or taking refresher courses.

### Instruments

#### Demographic questionnaire

Our demographic questionnaire collected basic information regarding the nurses—including sex, age, educational level, professional title, employment position, and years of work, ANTT-related training records, and attitude towards ANTT training.

#### ANTT knowledge questionnaire

Our ANTT knowledge questionnaire was designed based on a literature review and by referring to the ANTT Audit Tool and ANTT Competency Assessment Tool provided by the Association for Safe Aseptic Practices. The questionnaire included six core elements related to ANTT: key parts, key sites, no-touch technique, standard protection, critical aseptic fields, and general aseptic fields—for a total of 25 items. We scored 1 point for each correct answer and 0 points for incorrect ones to generate total scores ranging between 0 and 25 points (with higher scores indicating more ANTT knowledge). The Cronbach’s α value for this questionnaire was 0.674, indicating good internal consistency. The questionnaire used was self-developed for this study and thus has not been published elsewhere.

### Data collection

Data collection for this study was conducted using the “Wenjuanxing” professional electronic questionnaire survey platform, which allowed for anonymous completion. Before data collection, the purpose and significance of the survey were explained to the nursing department and head nurse of the ward before obtaining consent and determining the time of data collection. In January 2024, members of the research team explained the purpose and significance of the survey to nurses at the monthly meeting of each department using a unified guideline, distributed the questionnaire’s two-dimensional code, and answered questions related to completing the questionnaire on the spot to ensure a high response rate. To avoid duplication and ensure the quality of the questionnaire, the background of the Wenjuanxing was set as follows: (1) each WeChat user could only fill out the survey once; (2) surveys with any omitted items were not submittable; and (3) no modifications were permitted after submission. Any questionnaires that were filled out in < 1 min were rejected.

### Ethical considerations

Ethical approval was obtained from the Ethics Committee of Sir Run Run Shaw Hospital (No: 20,240,240). Before the study began, all of the participants were informed about the purpose and process of the study, and their participation was voluntary. The guidelines of the Declaration of Helsinki (World Medical Association, 2013) were followed throughout this study.

### Statistical analysis

Descriptive statistics such as mean, standard deviation (SD), frequency, and percentage were used to summarise the study cohort’s demographic data and ANTT knowledge scores. Shapiro–Wilk and Kolmogorov–Smirnov tests were used to assess and confirm the normality of the data distribution. Univariate analyses were conducted using t-tests and analysis of variance. Multivariate analyses were conducted using multivariate linear regression to explore the risk factors related to ANTT knowledge. All tests were two-tailed, and P values of < 0.05 were considered statistically significant. All statistical analyses were performed using SPSS software version 25.0 (IBM Corp., Armonk, NY, USA).

## Results

A total of 458 questionnaires were distributed, and 458 valid questionnaires were analysed, for an effective recovery rate of 100%.

### Demographic characteristics

#### Level of ANTT knowledge

In a context where ANTT has not yet been included in the curriculum of nursing training in China and the hospital we surveyed did not conduct explicit ANTT training, 21.4% of the nurses in our cohort had prior knowledge of ANTT. Of these, 51.0% had received a certain level of practical ANTT training. A total of 30.1% of our participants indicated that they did not require ANTT training. The overall correct response rate on the ANTT knowledge questionnaire was 49.7%. The lowest correct rates were observed for identifying the key part (3.3%) and key site (4.4%), whereas the highest rates were related to ANTT risk assessment (94.8%) and hand hygiene (93.7%)(Table 1). The lowest ANTT knowledge score was 4, the highest was 22, and the mean score was 12.4 ± 2.4.

**Table 1:** Accuracy of each item in the nurses regarding ANTT Knowledge Questionnaire

Items	Accuracy
**Judgment question**	
1. Equipment can not be contaminated by Airborne during aseptic technique.	79.5%
2. Sterile means free from ALL living microorganisms.	37.7%
3. Asepsis means free from pathogenic micro-organisms in sufficient quantity to cause an infection.	85.4%
4.Wound and puncture site are Key-Parts.	3.3%
5. Syringe tips, sterile gauze, needles are Key-Sites.	4.4%
6. Procedure duration is not the practice variables should be considered in ANTT Risk Assessment.	66.2%
7.user competency is not the practice variables should be considered in ANTT Risk Assessment.	85.8%
8. ANTT requires non-sterile gloves to be worn when touching Key-Sites.	47.6%
9. Alcohol gel can be used for hand cleaning.	60.9%
10. If gloves were worn, hand cleaning is not required.	93.7%
**Single-selected question**	
11. the microbiological aim of ANTT is? (A) Sterile (B) Asepsis (C) Clean	70.1%
12. About ANTT, which statement is incorrect? A. A specific and well-defined type of aseptic technique B. Suitable for any healthcare organization, including community and nursing homes C. Suitable for all invasive clinical procedures D. In hospitals of different sizes, ANTT procedures can be different	34.1%
13. What are the two types of ANTT? A. Standard-ANTT and Strict-ANTT B. Standard-ANTT and Surgical-ANTT C. Standard-ANTT and Normative-ANTT D. Standard-ANTT and Specific-ANTT	27.7%
14. What is the fundamental practice concept that ANTT is based upon? A. Strict hand hygiene B. Protection of General Aseptic Field and Micro Critical Aseptic Field C. Protection of Key-Site and Key-Part D. Protection of General Aseptic Field and Critical Aseptic Field	16.3%
15. Which is the right definition of a Key-Part? A. The part of the procedure equipment that, if contaminated, is likely to contaminate the patient B. Any portal of entry into the patient	81.4%
16. which practice variables should be considered in ANTT Risk Assessment? A. Procedure invasiveness B. The number and size of Key-Parts C. Any relevant environmental factors	94.8%
17. Which of the following is not the time for hand cleaning? A. In the ward corridor before entering the patient’s room B. Before touching the patient C. Before touching items around the patient (e.g., nightstands, linens, infusion sets, etc.) D. Clean/asepsis pre-procedure (e.g., placement of nasal tubes, insertion of catheters, blood draws, etc.) E. Before wearing gloves	53.7%
18. Which is not the personal protective equipment? A. Clean gloves B. Sterile gloves C. Sterile drape D. Sterile tray	52.6%
19. In Standard-ANTT, Key-Parts(e.g. syringe tip)should be protected by which type of aseptic field? A. General Aseptic Field B.Critical Aseptic Field C. Micro Critical Aseptic Field	32.1%
20. In Surgical-ANTT, Key-Parts(e.g. needles)should be protected by which type of aseptic field? A. General Aseptic Field B.Critical Aseptic Field C. Micro Critical Aseptic Field	67.7%
21. Which aseptic field of the ANTT does the procedure pack belong to? A. General Aseptic Field B.Critical Aseptic Field C. Micro Critical Aseptic Field	38.4%
22. Which aseptic field of the ANTT does the aseptic drape belong to? A. General Aseptic Field B.Critical Aseptic Field C. Micro Critical Aseptic Field	39.5%
23. Which aseptic field of the ANTT does the inside of packaging belong to? A. General Aseptic Field B.Critical Aseptic Field C. Micro Critical Aseptic Field	32.3%
24. Which procedure should use a standard-ANTT? A. Central venous catheterization B. Maintenance of vascular access devices C. Surgeries D. Urinary catheterization	24.9%
25. The following can be used as reasons for choosing a standard-ANTT for procedure? A. Procedure duration is less than 20 min B. invasiveness of Procedure C. Key- Site protection can be achieved by using a sterile drape D. Wear personal protective equipment during procedure	13.3%

#### Univariate analysis

The results of our univariate analysis indicated that clinical nurses with different professional titles, educational levels, years of work, prior knowledge of ANTT, training experience in ANTT, and training needs regarding ANTT exhibited statistically significant differences in their ANTT knowledge scores (*P* < 0.05). By contrast, there were no significant differences in the ANTT knowledge scores among clinical nurses based on sex, department, position, or age. These findings are summarised in Table 2.

**Table 2:** Sociodemographic characteristics correlation with ANTT knowledge scores

Variable	Frequency (%)/ Mean(SD)	ANTT score($(\bar x\, \pm \,{\text{S}})$±S)	F/t	*P*
**Age(years)**	30.1(6.9)	12.4 ± 2.5	0.062	0.186
**Sex**			0.719	0.456
Male	20(4.4%)	12.1 ± 2.1		
Female	438(95.6)	12.4 ± 2.4		
**Department**			0.923	0.497
internal medicine	185(40.4)	12.4 ± 2.5		
surgery	126(27.5)	12.2 ± 2.6		
Emergency/intensive care unit	101(22.1)	12.5 ± 2.2		
operating room and othersa	46(10.0)	12.9 ± 1.8		
**Professional title**			4.586	0.004
Jonior entry-level nurse	144(31.4)	12.2 ± 2.3		
Senior nurse	88(19.2)	12.2 ± 2.3		
Assistant chief nurse	222(48.5)	12.6 ± 2.5		
Associated chief nurse	4(0.9)	16.3 ± 2.4		
**Work position**			2.035	0.108
Clinical nurse	411(89.8)	12.4 ± 2.4		
Educational Nurses	23(5.0)	12.7 ± 2.8		
Specialist nurses	12(2.6)	12.7 ± 1.6		
Head nurses	12(2.6)	14.1 ± 2.7		
**Educational level**			3.868	0.022
College degree	3(0.7)	10.7 ± 1.5		
Bachelor degree	422(92.1)	12.4 ± 2.4		
Master degree	33(7.2)	13.4 ± 2.9		
**Years of experience**			3.309	0.020
≤1	133(29.0)	12.4 ± 2.3		
1 to 2	25(5.5)	11.2 ± 2.5		
3 to 5	65(14.2)	12.1 ± 2.4		
≥ 5	235(51.3)	12.7 ± 2.5		
**Prior knowledge of ANTT**			14.108	< 0.001
Yes	98(21.4)	13.2 ± 2.6		
No	360(78.6)	12.2 ± 2.4		
**Have received training related to ANTT**			5.033	0.007
Yes	50(10.9)	13.5 ± 2.5		
No	400(87.3)	12.3 ± 2.4		
Don’t know	8(1.8)	12.3 ± 2.5		
**Need training on ANTT**			13.817	< 0.001
Yes	320(69.9)	12.7 ± 2.4		
No	138(30.1)	11.8 ± 2.4		

Note: ^a^Others include haemodialysis room and endoscopy room

#### Multivariate linear analysis

Multivariate analysis was performed using a multivariate linear regression model, with the ANTT knowledge score as the dependent variable. The independent variables were those that exhibited statistically significant differences in the univariate analysis. The results showed that professional title, educational level, and training needs regarding ANTT were independently associated with the level of ANTT knowledge among nurses (Table 3).

**Table 3:** Results of multiple linear regression analysis

Variable	B-Value	Standard Error	Standard regression coefficient	t	*p*
Constant	12.610	1.133		11.131	0.000
Work position	0.779	0.344	0.285	2.261	0.024
Education	1.002	0.420	0.112	2.384	0.018
Years of experience	-0.355	0.235	-0.190	-1.509	0.132
Have prior knowledge of ANTT					
Yes	Ref				
No	-0.568	0.319	-0.095	-1.779	0.076
Have received training related to ANTT					
Yes	Ref				
No	-0.420	0.375	-0.059	-1.121	0.263
Need training on ANTT					
Yes	Ref				
No	-0.700	0.248	-0.132	-2.819	0.005

## Discussion

### Nurses possess inadequate knowledge regarding ANTT

ANTT represents one of the most important clinical competencies in healthcare. Understanding it lays the foundation for nurses to apply its approach in clinical settings. This emphasises the practical ability of clinical nurses to perform ANTT, which are directly linked to patient safety and play significant roles in the prevention and control of hospital infections. The results of this study indicated that the overall correct response rate of our cohort of nurses to the ANTT knowledge questionnaire was 49.7%. Additionally, only 21.4% of the nurses had prior knowledge of ANTT, indicating a significant lack of knowledge regarding this topic. This deficiency may be attributable to the insufficient dissemination of ANTT in China. Since the late 1990s, when ANTT was first developed, it has been successively adopted by many countries, a few of which have even included it in their national regulations. However, it was only after it was included in the Infusion Therapy Standards of Practice in 2021 that nurses in China began to gradually become aware of ANTT. As a result, many nurses in China currently have limited awareness and understanding of ANTT.

ANTT is a standardised approach to aseptic technique that has now been globally recognised and has become the standard aseptic technique used in hospitals across Australia, the United Kingdom, and 15 other countries. In these countries, ANTT is used in diverse fields, including wound healing and intravenous therapy. Nevertheless, recent research conducted in these regions has highlighted the need to enhance ANTT expertise among nurses. In wound care practice, although ANTT has been included in national standards for some time, one study reported that fifty-nine (50.4%) of respondents reported being unaware of the national standards pertaining to wound management [15]. An observational study examining nursing practices related to preventing postoperative wound infections revealed a notable disparity between the use of gloves and evidence-based guideline recommendations [16]. This significant variance reflects limited awareness of ANTT guidelines among nurses, which aligns with the results reported by Gillespie et al. [15]. However, despite continuous research and attention paid to aseptic techniques in China, the study of ANTT remains in its infancy. Moreover, ANTT has yet to gain widespread acceptance as a standard approach within the clinical realm, and hospitals have yet to initiate specific training programmes pertaining to it. As a result, many nurses in China lack sufficient awareness regarding ANTT.

### Limited awareness regarding the significance of ANTT

In this study, 138 clinical nurses believed that they did not require ANTT training, indicating a lack of awareness concerning the significance of this technique among the cohort. The Chinese government places significant emphasis on preventing and controlling infections across all hospitals and has issued relevant policies and programmes to encourage hospitals to strengthen their implementation of prevention and control measures to combat hospital infections [17, 18]. However, ANTT, as a fundamental skill for infection prevention and control, has not received as much attention in China as it has in other countries such as the United Kingdom and Australia. This may lead to limited awareness among nurses regarding the significance of ANTT. Therefore, there is an urgent need to promote and implement ANTT at both the government and hospital levels.

The Chinese government should further improve and implement relevant policies and regulations, taking ANTT as an important measure for infection prevention and control and promoting its clinical implementation. Hospitals should proactively align themselves with governmental policies regarding hospital infection prevention and control. To achieve this, efforts must be initiated at three distinct levels: knowledge, attitude, and action. First, nurse education must be enhanced at the knowledge level. Nurses should then be assisted with regard to cultivating a correct attitude towards ANTT, enabling them to appreciate its crucial role in clinical practice. The ultimate objective should be to improve the clinical application of ANTT and enhance its infection prevention and control capabilities.

### The contributions of professional title and educational level to ANTT knowledge

The results of this study showed that Master’s degrees and associated chief nurses were observed to have the highest ANTT knowledge scores. Conversely, those with the professional title of Jonior entry-level and Senior nurse and college degrees had the lowest scores. Professional title and educational level were independently associated with the level of ANTT knowledge. The professional titles of nurses are closely linked to their resources. Those with senior professional titles tend to have extensive clinical experience and more resources and opportunities for learning. Moreover, professional titles serve as gauges of an individual’s overall capabilities. Advancement to a senior title requires passing examinations, publishing scientific articles, and declaring a subject of specialisation—thus indicating that senior-title nurses possess deeper knowledge reserves and scientific research literacy. Although educational level is associated with personal learning ability and knowledge, higher educational levels often equate to stronger learning abilities [19] and broader knowledge bases. Several studies have demonstrated that professional title and educational level influence levels of knowledge among nurses [20–22]. Therefore, nursing managers should prioritise addressing knowledge gaps among nurses with junior and intermediate titles and those with limited educational levels. Examining the barriers to and enablers of knowledge acquisition among this group is expected to enable the development of targeted strategies to enhance ANTT-related knowledge. In addition, fostering clinical nurses’ learning capabilities and encouraging them to pursue higher educational levels and professional titles is likely to enhance their knowledge in multiple dimensions.

### Enhancing ANTT knowledge through sustained, multi-form, and stratified training

Training represents another important way to enhance knowledge and clinical competence in nurses [23, 24]. Our study reported that 69.9% of nurses required ANTT training, making it imperative for nursing managers to implement this type of training for their staff as soon as possible. Furthermore, various training formats can enhance effectiveness, including mobile electronic platform-based [25], step-by-step [26, 27], game-based [28], and simulation-based training [29].

Bharathi et al. [29] used simulation-based training methods for all staff nurses and junior resident doctors in a tertiary-care neonatal unit, which was able to significantly improve performance on the ANTT test. Chen et al. [30] enhanced the effectiveness of training and improved learning satisfaction through multiform training. However, the impact of training may diminish over time. A study examining ANTT training for medical students reported that test scores significantly decreased 10 weeks after an ANTT training session, compared to immediately following the completion of the training [31]. Therefore, when implementing ANTT training, it is essential to circumvent the weakening effect of time on the effectiveness of a single training session. Sustained multiform training should be conducted to enhance the effectiveness of training and enable the level of ANTT knowledge to improve steadily.

In addition, the influence of professional titles on knowledge level suggests that the knowledge and experience of senior-level nurses should be leveraged when carrying out stratified ANTT training. Typically, a higher title corresponds to more years of experience, and senior nurses often serve as excellent role models for younger ones. Hospitals can start training senior nurses with mid- or high-level titles first, to improve their clinical abilities regarding ANTT, after which these nurses can pass on their knowledge and skills to more junior ones, ultimately enhancing the overall skillset of the nursing team.

To cater to the diverse requirements of nurses, it is imperative to develop tailored training programmes and establish a sustained, multi-form, and stratified training system aimed at enhancing understanding of ANTT in nurses.

### Limitations

While this study offered insightful information regarding the present understanding of ANTT knowledge and related factors in nurses, it is important to acknowledge its limitations. First, the sample enrolled in this study was sourced solely from a single centre; thus, the representativeness of the sample was limited. Besides, there is no universal ANTT knowledge scale, the scale used in this study was designed by the researcher herself with referencing the ANTT Audit Tool and ANTT Competency Assessment Tool, so the research instrument has limitations. Further more, owing to the cross-sectional nature of the study, it was not feasible to investigate the causal relationship between ANTT knowledge and the various factors that may influence it. To gather evidence supporting the promotion and application of ANTT, experimental multi-centre studies should be conducted in the future.

## Conclusion

Awareness of ANTT and its importance is currently insufficient among nurses working in Zhejiang Province, China. Their levels of ANTT-related knowledge appear to be associated with professional titles, educational levels, and training needs. As a standardised aseptic technique, the promotion and application of ANTT in the clinic are of great significance for preventing hospital infections and enhancing the overall quality of healthcare. Therefore, it is imperative to initiate support for clinical nurses to develop accurate cognition of ANTT at multiple levels—including the state, hospital, and individual levels. Sustained, multi-form and stratified training should be conducted while leveraging the leadership roles of experienced nurses as examples for mentoring more junior ones. These approaches are expected to lead to a comprehensive increase in the knowledge base of nurses who practice ANTT, thereby enhancing its clinical application. Besides, whether ANTT training at the medical school is effective in increasing the overall knowledge of ANTT in the nurse population could be explored in future studies.

## Data Availability

All data and materials are included in this published article.

## Abbreviations

HAIs: Healthcare associated infections
ANTT: Aseptic non touch technique
SD: Standard deviation
